# Development and internal validation of a machine-learning-based risk identification model for diabetic kidney disease in patients with type 2 diabetes

**DOI:** 10.3389/fendo.2026.1888060

**Published:** 2026-07-28

**Authors:** Jianquan Luo, Guoxin Chen, Jiacheng Yang

**Affiliations:** Department of Endocrinology Laboratory, Sihui People’s Hospital, Sihui, Guangdong, China

**Keywords:** diabetic kidney disease, machine learning, non-invasive screening, risk prediction model, SHAP interpretability, type 2 diabetes mellitus

## Abstract

**Background:**

Diabetic kidney disease (DKD) is a major microvascular complication of type 2 diabetes mellitus (T2DM) and the leading cause of end-stage renal disease in China. Limited disease awareness and insufficient early screening tools hinder timely intervention for DKD patients. This study aimed to develop and internally validate a non-invasive machine learning-based prediction model for early DKD risk identification among T2DM patients using routine clinical laboratory indicators.

**Methods:**

A retrospective cross-sectional study was conducted with 602 eligible T2DM patients (457 without DKD, 145 with DKD) recruited from Sihui People’s Hospital between January 2023 and June 2024. Subjects were randomly split into an 8:2 training set (n=481) and independent test set (n=121). Baseline clinical characteristics were compared between groups. Univariate and multivariate logistic regression analyses were performed to screen independent DKD predictors. Six machine learning algorithms including logistic regression, XGBoost, random forest, AdaBoost, support vector classifier (SVC), and Gaussian naive Bayes (GNB) were constructed and comprehensively assessed via AUC, accuracy, sensitivity, specificity, calibration curves, decision curve analysis (DCA), and SHapley Additive exPlanations (SHAP) interpretability analysis.

**Results:**

Baseline comparisons showed that DKD patients presented worse glycolipid, hepatic, renal, hematological and urinary protein indicators than patients with isolated T2DM, while sex, age, BMI, DBP and FPG showed no significant intergroup differences (all P>0.05). Multivariate logistic regression identified glycated hemoglobin (HbA1c), β_2_-microglobulin (β_2_MG), and urine protein (PRO) as independent risk factors, while serum albumin (ALB) and estimated glomerular filtration rate (eGFR) acted as protective factors. Single indicator ROC analysis showed PRO and β_2_MG achieved the highest diagnostic AUC of 0.944. All six machine learning models exhibited excellent discriminative performance with validation AUCs over 0.975. Logistic regression was selected as the optimal model, yielding a test-set AUC of 0.979, sensitivity of 89.7%, and specificity of 92.4%. The model demonstrated favorable calibration and sustained positive net clinical benefit across almost all threshold probabilities in DCA. SHAP analysis ranked HbA1c, ALB, PRO, eGFR, and β_2_MG as the top five predictive features, clarifying the individual risk contribution of each biomarker.

**Conclusions:**

The interpretable logistic regression model built on routine non-invasive clinical indicators reliably identifies DKD risk in T2DM patients. Glycemic control and renal injury biomarkers serve as core predictive factors. This tool provides convenient, low-cost early risk stratification for clinical practice, especially for primary care settings. Further external multicenter prospective validation is required to generalize its clinical application.

## 1 Introduction

Diabetic kidney disease (DKD) is a frequent and severe microvascular complication of diabetes and a major cause of end-stage renal disease (ESRD) (1). With the increasing prevalence of diabetes, DKD imposes a growing health and economic burden, particularly in rapidly aging populations such as China. In China, chronic kidney disease due to type 2 diabetes (CKD-T2D) has overtaken glomerulonephritis as the leading cause of CKD (2). A systematic literature review of Chinese DKD patients reported suboptimal metabolic control (mean HbA1c 8.4%), high comorbidity burdens (hypertension 65.0%, dyslipidemia 50.2%), and alarmingly low disease awareness (<20%) and early treatment rates (<50%) (3). Therefore, the development of non-invasive, accurate, and easy-to-implement risk prediction tools is of urgent clinical need.

DKD risk evaluation involves multiple clinical domains, including glycemic control, renal function, and metabolic indicators. However, single markers often fail to fully capture disease risk, and conventional statistical models may be limited in handling multidimensional features and complex relationships among predictors (4). Machine learning approaches, with their capacity for nonlinear modeling and interaction detection, have increasingly been applied to individualized risk assessment and prediction modeling (5).

For clinical translation, a model must not only have high discrimination but also be well calibrated, clinically useful, and interpretable. Decision curve analysis (DCA) quantifies net benefit across decision thresholds (6), while SHAP (SHapley Additive exPlanations) can attribute individual predictions to features, reducing the “black-box” barrier and supporting clinical trust (7).

Therefore, using real-world electronic medical records, we integrated Logistic regression feature selection and machine learning methods to develop and validate a non-invasive DKD prediction/classification model for T2DM patients, and evaluated discrimination, calibration, clinical utility, and interpretability using ROC/AUC, calibration curves, DCA, and SHAP analyses.

## 2 Methods

### 2.1 Study design and setting

#### 2.1.1 Study data sources

This was a retrospective cross−sectional study conducted at Sihui People’s Hospital (Guangdong, China), using medical records from January 2023 to June 2024.

#### 2.1.2 Ethics approval and consent to participate

The study was approved by the Medical Ethics Committee of the People’s Hospital of Sihui (approval number: 2026_FSMY_10011) and conducted in accordance with the Declaration of Helsinki. Given the retrospective cross−sectional design and minimal risk, the ethics committee granted a waiver of informed consent.

#### 2.1.3 Participants

Inclusion criteria: (1) Patients met the diagnostic criteria for type 2 diabetes mellitus (T2DM) specified in the *Chinese Guidelines for the Prevention and Treatment of Type 2 Diabetes (2020 Edition)* (8), with an age of no less than 18 years old; (2) For patients with multiple hospital admissions within one year, only the data from the first admission was enrolled; (3) Patients assigned to the DKD group were clearly diagnosed via the clinical electronic medical record system and satisfied the diagnostic criteria outlined in the Expert Consensus on the Diagnosis and Treatment of Diabetic Kidney Disease (2019 Edition) (9); (4) Patients in the isolated T2DM group had no clinical evidence of diabetic kidney disease (DKD); (5) All enrolled patients had complete clinical and laboratory examination data available.

Exclusion criteria: (1) type 1 diabetes or other specific types of diabetes; (2) other primary kidney diseases (e.g., glomerulonephritis, interstitial nephritis, obstructive nephropathy); (3) acute kidney injury or recent treatment affecting renal function; (4) severe infection, malignancy, pregnancy, or other systemic diseases affecting renal function; (5) severe hepatic insufficiency, cardiopulmonary dysfunction, or advanced malignant tumors; (6) key variables missing, making statistical analysis impossible.

**Figure 1:**
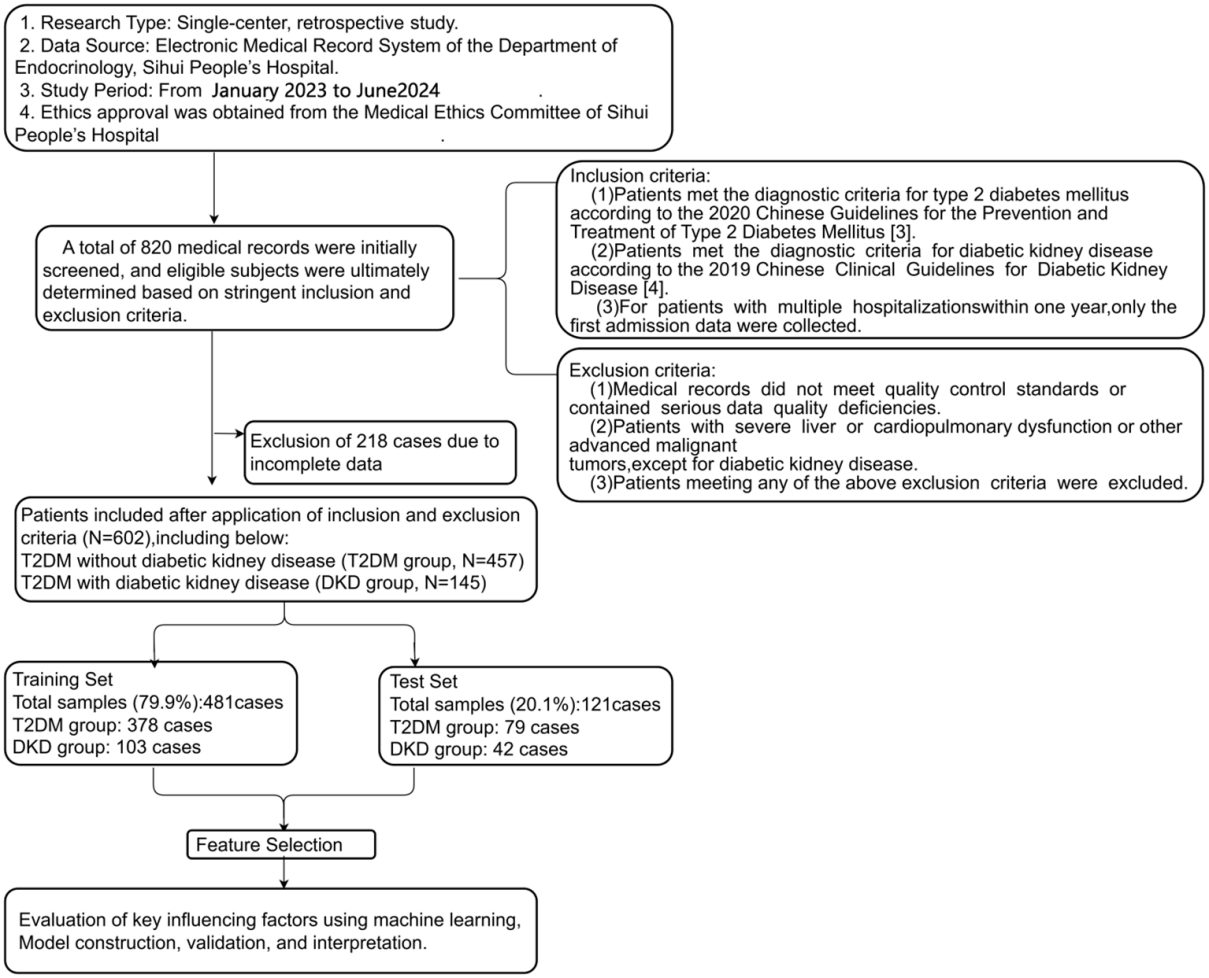
Patient screening and analysis flowchart (as described).

An initial screening of 820 medical records was performed. After applying the above criteria and data quality control, 602 patients were finally included: 457 in the T2DM alone group and 145 in the DKD group. The patient screening and grouping process is illustrated in **Figure 1**.

### 2.2 Data collection and preprocessing

Electronic medical records provided demographic characteristics (age, sex, body mass index [BMI]), vital signs, and 19 candidate laboratory indicators: systolic blood pressure (SBP), diastolic blood pressure (DBP), fasting plasma glucose (FPG), 2−hour postprandial glucose (2h−PG), glycated hemoglobin (HbA1c), fructosamine (FMN), alanine aminotransferase (ALT), aspartate aminotransferase (AST), gamma−glutamyl transferase (GGT), low−density lipoprotein cholesterol (LDL−C), high−density lipoprotein cholesterol (HDL−C), triglycerides (TG), total cholesterol (TC), albumin (ALB), globulin (GLB), β_2_−microglobulin (β_2_−MG), estimated glomerular filtration rate (eGFR), urine protein (PRO), and hemoglobin (HGB).

All laboratory measurements were performed using standardized automated analyzers (Beckman AU6800 for biochemistry, Hitachi for HbA1c, Beckman for urinalysis) with dedicated reagents. Daily internal quality control results consistently met required standards. Missing values with a rate below 5% were processed according to variable data type, missing rate and inter-variable correlation. Mode imputation was applied to categorical variables, mean imputation was adopted for normally distributed continuous variables, and k-nearest neighbor (KNN) imputation was performed for the remaining numeric variables with correlated features to maximally preserve the original data distribution and reduce imputation bias.

### 2.3 Feature selection

Univariate and multivariate logistic regression analyses were performed for optimized screening of candidate variables to construct the prediction model. Variables with statistical significance in univariate logistic regression were included in the multivariate regression analysis to screen independent predictors, reduce data dimensionality and avoid model overfitting.

### 2.4 Data split and internal validation

All enrolled participants were randomly divided into a training set (n=481, including 378 patients with type 2 diabetes mellitus [T2DM] and 103 patients with diabetic kidney disease [DKD]) and an independent test set (n=121, including 79 T2DM patients and 42 DKD patients) at a ratio of 8:2. Five-fold cross-validation was performed to determine the optimal regularization parameter λ, defined as the value corresponding to the minimum mean squared error.

#### 2.4.1 Modeling algorithms

Based on the predictive variables screened by univariate and multivariate logistic regression analysis, six classification machine learning models were established and systematically compared, including logistic regression, extreme gradient boosting (XGBoost), random forest, AdaBoost, Gaussian naive Bayes (GNB), and support vector classifier (SVC).

#### 2.4.2 Evaluation metrics

A multi-dimensional evaluation system was adopted to comprehensively assess model performance. Model discriminative ability was evaluated using receiver operating characteristic (ROC) curves and the corresponding area under the curve (AUC) with 95% confidence intervals (CIs). Calibration curves were plotted to verify the consistency between predicted and actual outcomes. Decision curve analysis (DCA) was performed to quantify the clinical net benefit and practical utility of the model. Additionally, SHAP analyses, including summary plots, feature importance bar plots, and force plots, were applied to interpret model interpretability and clarify the contribution of each predictive feature.

### 2.5 Statistical analysis

All data were analyzed using SPSS 27.0 and Python (scikit−learn, XGBoost, SHAP). Normally distributed continuous variables were reported as mean ± SD and compared by Student’s t−test; non−normally distributed continuous variables were reported as median (Q1, Q3) and compared by Mann−Whitney U test. Binary categorical variables were reported as n (%) and compared using χ² tests. Statistical significance was set at p<0.05.

## 3 Results

### 3.1 Baseline characteristics

A total of 602 patients were enrolled in this study, including 457 patients with type 2 diabetes mellitus (T2DM) alone and 145 patients with diabetic kidney disease (DKD). No statistically significant differences were found between the two groups in terms of sex, age, body mass index (BMI), diastolic blood pressure (DBP) and fasting plasma glucose (FPG) (all P>0.05). Compared with the T2DM alone group, the DKD group exhibited significantly higher levels of 2h-PG, HbA1c, FMN, ALT, AST, GGT, LDL-C, TG, TC, β_2_MG and PRO, as well as significantly lower levels of eGFR, ALB, GLB and HGB (all P<0.05). No significant intergroup difference was observed in HDL-C (**Table 1**).

**Table 1:** Baseline clinical characteristics of participants.

Variables	Overall (n=602)	T2DM group (n=457)	DKD group (n=145)	Statistic	P-value	Analytical methods
Sex,n(%)				2.752	0.097	Chi-square test
Male	321 (53.32)	235 (51.42)	86 (59.31)			
Female	281 (46.68)	222 (48.58)	59 (40.69)			
Age,years,median[IQR]	62 [47, 71]	63 [50, 71]	59 [35, 70]	1.788	0.074	Mannwhitney-U
BMI,median[IQR]	22.2 [19.3, 25.2]	22.2 [19.3, 25.2]	22.2 [20.4, 24.8]	−0.354	0.723	Mannwhitney-U
SBP,mmHg,median[IQR]	143 [130, 159]	141 [128, 157]	145 [135, 166]	−2.786	0.005	Mannwhitney-U
DBP,mmHg,median[IQR]	84 [76, 92]	83 [75, 92]	84 [78, 92]	−0.729	0.466	Mannwhitney-U
FPG,mmol/L,median[IQR]	10.08 [7.94, 14.11]	9.98 [7.92, 13.72]	10.65 [8.19, 17.32]	−1.934	0.053	Mannwhitney-U
2h-PG,mmol/L,median[IQR]	7.56 [5.54, 12.47]	6.36 [5.12, 8.35]	19.07 [12.91, 23.49]	−15.942	<0.001	Mannwhitney-U
HbA1c,%,median[IQR]	7.1 [6.1, 8.5]	6.6 [5.9, 7.63]	9.6 [7.6, 11.5]	−11.711	<0.001	Mannwhitney-U
FMN,mmol/L,median[IQR]	1.98 [1.69, 2.26]	1.88 [1.62, 2.06]	2.39 [2.06, 2.87]	−10.211	<0.001	Mannwhitney-U
ALT,U/L,median[IQR]	16 [11, 25]	14 [10, 20]	35 [23, 77]	−13.665	<0.001	Mannwhitney-U
AST,U/L,median[IQR]	19 [15, 27]	18 [14, 21]	35 [25, 62]	−14.600	<0.001	Mannwhitney-U
GGT,U/L,median[IQR]	27 [17, 41]	23 [15, 32]	52 [33, 94]	−11.486	<0.001	Mannwhitney-U
LDL-C,mmol/L,median[IQR]	2.74 [2.05, 3.63]	2.45 [1.91, 3.05]	4.15 [3.24, 5.13]	−12.905	<0.001	Mannwhitney-U
HDL-C,mmol/L,median[IQR]	1.2 [0.98, 1.47]	1.27 [1.08, 1.54]	0.91 [0.73, 1.06]	11.664	<0.001	Mannwhitney-U
TG,mmol/L,median[IQR]	1.41 [0.93, 2.29]	1.22 [0.82, 1.70]	2.83 [2.01, 4.32]	−13.153	<0.001	Mannwhitney-U
TC,mmol/L,median[IQR]	4.55 [3.61, 5.70]	4.13 [3.41, 4.97]	6.36 [5.38, 7.86]	−13.221	<0.001	Mannwhitney-U
β_2_MG,mg/L,median[IQR]	2.27 [1.63, 4.30]	1.94 [1.51, 2.60]	7.84 [4.56, 19.29]	−16.107	<0.001	Mannwhitney-U
ALB,g/L,median[IQR]	41.8 [36.6, 44.6]	43.2 [40.4, 45.3]	31.0 [25.5, 35.1]	16.119	<0.001	Mannwhitney-U
GLB,g/L,median[IQR]	29.9 [26.0, 33.6]	31.3 [28.2, 34.9]	24.7 [22.0, 27.1]	13.21	<0.001	Mannwhitney-U
eGFR,mL/min,median[IQR]	64.62 [47.00, 86.00]	67.00 [64.62, 92.00]	31.00 [10.00, 57.00]	13.138	<0.001	Mannwhitney-U
PRO,mg/dL,median[IQR]	0 [0, 50]	0 [0, 0]	102 [50, 300]	−15.991	<0.001	Mannwhitney-U
HGB,g/L,median[IQR]	133 [113, 147]	138 [126, 150]	92 [66, 112]	15.484	<0.001	Mannwhitney-U

Continuous variables are presented as median (interquartile range, IQR); categorical variables are expressed as n (%).

### 3.2 Univariate and multivariate logistic regression

Univariate analysis identified 20 variables significantly associated with DKD (p<0.05) (**Table 2**). Multivariate logistic regression revealed that HbA1c, β_2_-MG, and PRO were independent risk factors, while ALB and eGFR were protective factors (**Table 3**).

**Table 2:** Univariate logistic regression analysis of factors associated with DKD.

Predictor	OR	95% CI	P-value
Sex	1.006	0.967–1.046	0.768
Age	1.004	0.990–1.017	0.585
BMI	0.726	0.497–1.061	0.098
SBP	1.049	1.014–1.084	0.005
DBP	0.99	0.980–1.000	0.041
FPG	1.147	1.115–1.180	<0.001
2h-PG	1.651	1.489–1.830	<0.001
HbA1c	1.03	1.023–1.037	<0.001
FMN	1.841	1.642–2.064	<0.001
ALT	1.106	1.083–1.130	<0.001
AST	1.011	1.004–1.019	0.003
GGT	1.01	1.007–1.014	<0.001
LDL-C	1.402	1.328–1.480	<0.001
HDL-C	0.687	0.645–0.732	<0.001
TG	2.139	1.812–2.525	<0.001
TC	1.506	1.425–1.570	<0.001
β_2_MG	2.728	2.277–3.267	<0.001
ALB	0.015	0.007–0.036	<0.001
GLB	0.913	0.898–0.927	<0.001
eGFR	0.943	0.933–0.952	<0.001
PRO	7.597	4.933–11.699	<0.001
HGB	0.705	0.661–0.752	<0.001

OR, odds ratio; CI, confidence interval.

**Table 3:** Multivariate logistic regression analysis of independent predictors for DKD.

Predictor	Estimate	SE	OR (95%CI)	Z	P-value	VIF
Intercept	12.593	14.142	294343.09 (0.00–1.6×10²¹)	0.89	0.373	
FPG	−0.056	0.035	0.945 (0.865–1.001)	−1.62	0.105	
2h-PG	0.028	0.19	1.028 (0.745–1.336)	0.15	0.883	
HbA1c	1.437	0.581	4.207 (1.689–18.145)	2.47	0.013*	1.216
FMN	−1.269	1.595	0.281 (0.005–4.437)	−0.80	0.426	
ALT	0.064	0.056	1.066 (0.964–1.219)	1.13	0.257	
GGT	0.002	0.007	1.002 (0.989–1.018)	0.27	0.789	
LDL-C	0.969	1.877	2.634 (0.059–136.450)	0.52	0.606	
HDL-C	2.6	2.65	13.462 (0.095–4115.399)	0.98	0.327	
TG	0.803	0.516	2.231 (0.911–7.383)	1.56	0.12	
TC	−0.301	1.464	0.740 (0.038–16.160)	−0.21	0.837	
β_2_MG	0.021	0.01	1.022 (1.003–1.046)	2.09	0.036*	1.791
ALB	−1.397	0.592	0.247 (0.055–0.623)	−2.36	0.018*	2.295
GLB	−0.252	0.186	0.777 (0.494–0.927)	−1.36	0.176	
eGFR	−0.088	0.038	0.916 (0.830–0.974)	−2.33	0.02*	1.528
PRO	1.23	0.485	3.421 (1.568–12.035)	2.54	0.011*	1.919
HGB	0.11	0.19	1.116 (0.756–1.633)	0.58	0.564	

all VIF values were less than 5, suggesting no obvious multicollinearity among these independent predictors. *P<0.05 indicates statistically significant difference.

### 3.3 ROC analysis of single indicators

Among individual indicators, PRO and β_2_-MG showed the highest diagnostic performance (AUC = 0.944), followed by eGFR (0.939), and HbA1c (0.926). eGFR had the highest sensitivity (0.952), and FPG had the best specificity (0.838) (**Table 4**).

**Table 4:** ROC analysis of single laboratory indicators for DKD prediction.

Variable	AUC (95%CI)	Sensitivity (95%CI)	Specificity (95%CI)	J	Cutoff	Accuracy (95%CI)
FPG	0.902 (0.873–0.927)	0.821 (0.756–0.907)	0.838 (0.733–0.888)	0.659	24	0.829 (0.771–0.867)
2h-PG	0.864 (0.816–0.900)	0.788 (0.710–0.869)	0.793 (0.716–0.891)	0.581	27.9	0.800 (0.747–0.836)
HbA1c	0.926 (0.902–0.952)	0.893 (0.831–0.935)	0.814 (0.751–0.914)	0.707	116	0.873 (0.842–0.909)
FMN	0.862 (0.824–0.890)	0.829 (0.789–0.936)	0.766 (0.642–0.835)	0.595	59	0.819 (0.786–0.870)
ALT	0.876 (0.848–0.908)	0.759 (0.728–0.936)	0.834 (0.639–0.862)	0.592	23	0.791 (0.710–0.847)
GGT	0.781 (0.736–0.817)	0.710 (0.616–0.773)	0.766 (0.719–0.861)	0.476	2.06	0.757 (0.711–0.810)
LDL-C	0.855 (0.811–0.885)	0.738 (0.677–0.908)	0.829 (0.636–0.868)	0.567	3.39	0.796 (0.698–0.840)
HDL-C	0.821 (0.790–0.857)	0.783 (0.739–0.840)	0.752 (0.678–0.824)	0.535	1.07	0.779 (0.743–0.821)
TG	0.862 (0.824–0.889)	0.779 (0.707–0.900)	0.807 (0.697–0.871)	0.587	1.92	0.798 (0.746–0.847)
TC	0.864 (0.827–0.896)	0.793 (0.692–0.839)	0.803 (0.782–0.889)	0.596	5.2	0.816 (0.780–0.858)
β_2_MG	0.944 (0.922–0.959)	0.912 (0.843–0.959)	0.841 (0.767–0.922)	0.754	37.1	0.889 (0.850–0.919)
ALB	0.930 (0.915–0.962)	0.876 (0.845–0.975)	0.873 (0.766–0.895)	0.749	50	0.859 (0.806–0.895)
GLB	0.816 (0.778–0.846)	0.814 (0.617–0.859)	0.707 (0.678–0.865)	0.521	31	0.763 (0.704–0.820)
eGFR	0.939 (0.919–0.957)	0.952 (0.838–0.977)	0.792 (0.774–0.912)	0.744	8.79	0.854 (0.816–0.899)
PRO	0.944 (0.926–0.960)	0.869 (0.824–0.932)	0.893 (0.851–0.921)	0.762	3.58	0.886 (0.855–0.910)
HGB	0.822 (0.778–0.860)	0.738 (0.620–0.798)	0.790 (0.761–0.891)	0.528	7.7	0.792 (0.759–0.839)

Youden index (Abbreviations:J) and optimal cutoff (Cutoff).

### 3.4 Performance of the six machine learning models

Detailed performance on the training and validation sets is presented in **Tables 5**, **6**. All six models demonstrated excellent discriminative ability with validation AUCs >0.975. Logistic regression achieved the highest validation AUC (0.986 [SD = 0.004]), with favorable balanced sensitivity (0.931) and specificity (0.925). XGBoost yielded the highest validation accuracy (0.950), accompanied by robust sensitivity (0.924) and specificity (0.958). Random Forest had the highest specificity (0.969), while logistic regression and SVM shared the same sensitivity value of 0.931 in the validation cohort.

**Table 5:** Performance of six machine learning models in the training set (5-fold CV).

Model	AUC (SD)	Cutoff (SD)	Accuracy (SD)	Sensitivity (SD)	Specificity (SD)	PPV (SD)	NPV (SD)	F1 (SD)	Kappa (SD)
XGBoost	0.996(0.000)	0.359(0.080)	0.973(0.004)	0.976(0.006)	0.972(0.007)	0.918(0.019)	0.992(0.002)	0.946(0.008)	0.928(0.011)
logistic	0.987(0.001)	0.209(0.030)	0.938(0.012)	0.960(0.018)	0.931(0.022)	0.817(0.042)	0.987(0.006)	0.882(0.018)	0.840(0.027)
RandomForest	1.000(0.000)	0.584(0.048)	1.000(0.000)	1.000(0.000)	1.000(0.000)	1.000(0.000)	1.000(0.000)	1.000(0.000)	1.000(0.000)
AdaBoost	0.995(0.001)	0.465(0.025)	0.965(0.009)	0.972(0.008)	0.963(0.014)	0.894(0.035)	0.991(0.003)	0.931(0.016)	0.908(0.022)
SVM	0.982(0.002)	0.495(0.083)	0.947(0.009)	0.952(0.004)	0.945(0.012)	0.848(0.030)	0.984(0.001)	0.896(0.015)	0.861(0.021)
GNB	0.978(0.001)	0.022(0.010)	0.932(0.011)	0.953(0.016)	0.925(0.019)	0.804(0.035)	0.984(0.005)	0.871(0.017)	0.826(0.025)

**Table 6:** Performance of six machine learning models in the validation set (5-fold CV).

Model	AUC (SD)	Cutoff (SD)	Accuracy (SD)	Sensitivity (SD)	Specificity (SD)	PPV (SD)	NPV (SD)	F1 (SD)	Kappa (SD)
XGBoost	0.984(0.004)	0.359(0.080)	0.950(0.015)	0.924(0.074)	0.958(0.020)	0.880(0.045)	0.976(0.022)	0.899(0.033)	0.866(0.042)
logistic	0.986(0.004)	0.209(0.030)	0.927(0.029)	0.931(0.044)	0.925(0.048)	0.815(0.102)	0.978(0.013)	0.863(0.043)	0.814(0.063)
RandomForest	0.985(0.004)	0.584(0.048)	0.939(0.024)	0.841(0.121)	0.969(0.021)	0.903(0.053)	0.952(0.034)	0.864(0.065)	0.825(0.078)
AdaBoost	0.982(0.006)	0.465(0.025)	0.944(0.012)	0.917(0.077)	0.952(0.023)	0.864(0.047)	0.974(0.023)	0.886(0.028)	0.849(0.035)
SVM	0.976(0.008)	0.495(0.083)	0.937(0.017)	0.931(0.038)	0.939(0.033)	0.838(0.075)	0.978(0.011)	0.878(0.026)	0.836(0.038)
GNB	0.976(0.011)	0.022(0.010)	0.919(0.031)	0.917(0.041)	0.919(0.049)	0.797(0.094)	0.973(0.013)	0.848(0.045)	0.793(0.066)

**Figure 2:**
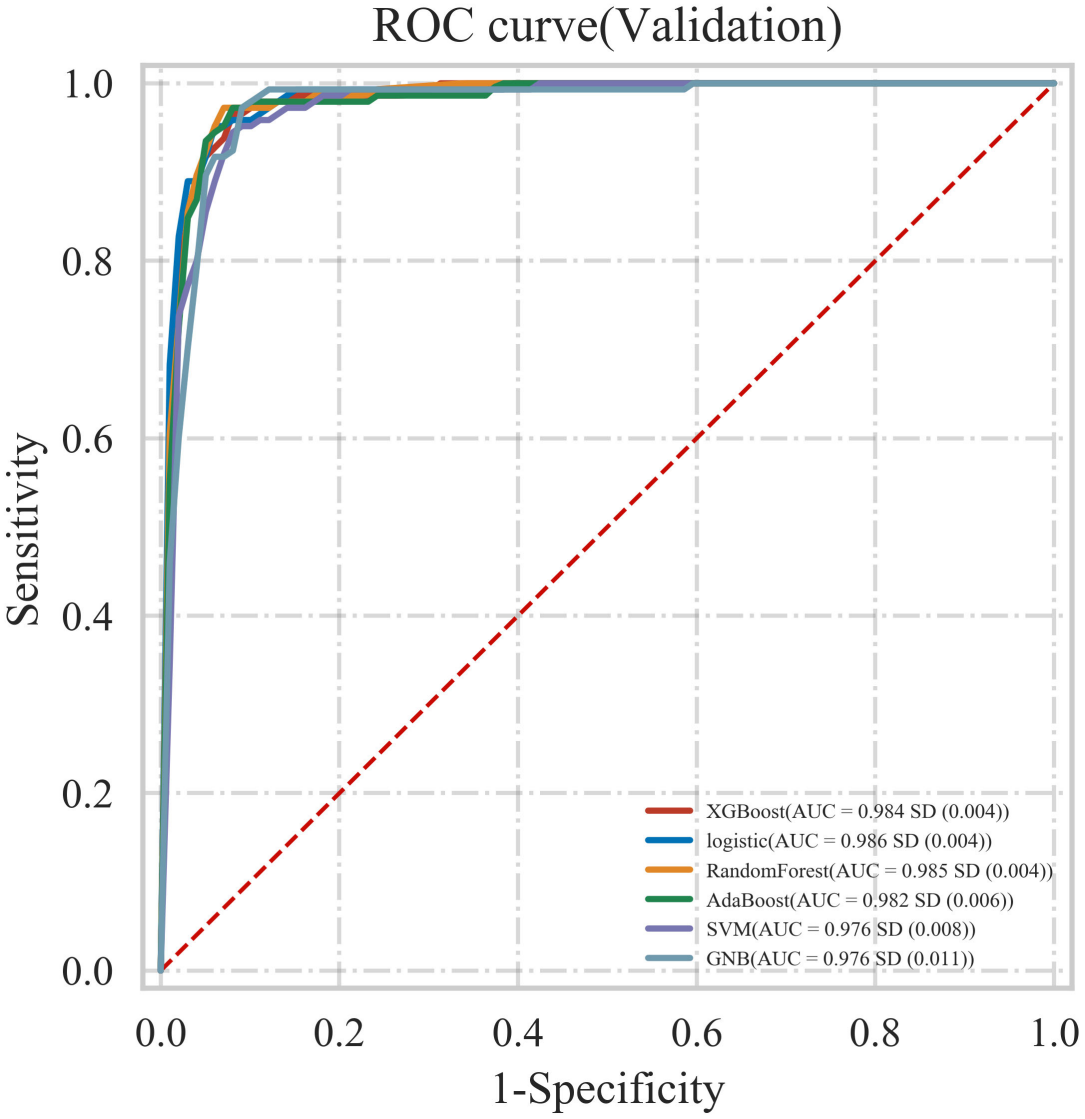
ROC curves of six models for DKD prediction. Six models achieved excellent predictive performance with area under the curve (AUC) values ranging from 0.976 to 0.986, indicating strong discriminative power to distinguish patients at risk of DKD from those without renal impairment.

**Figure 3:**
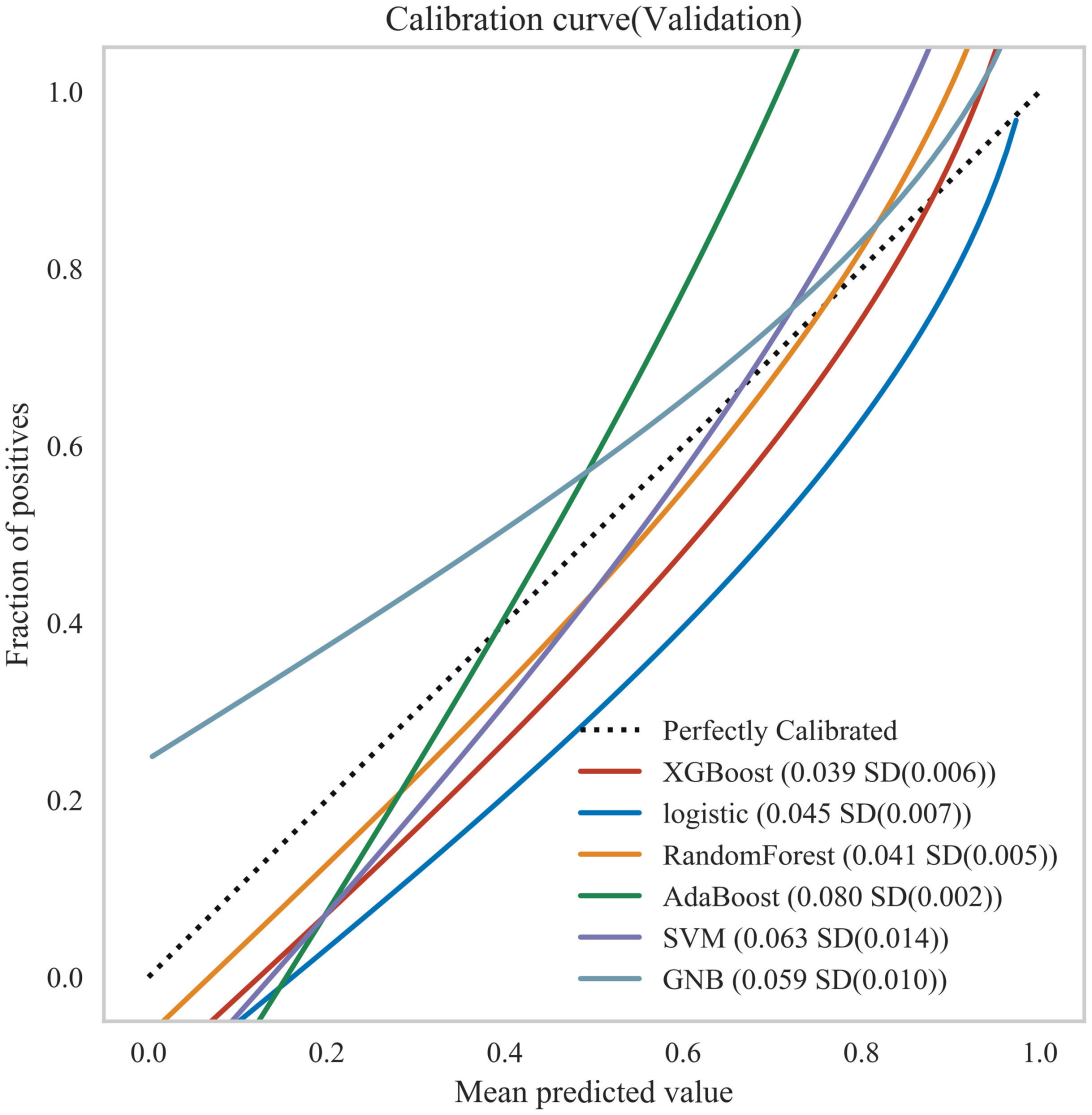
Calibration curve of the logistic regression model for predicting diabetic kidney disease risk. Demonstrated excellent calibration for Logistic regression (Brier score = 0.045, SD = 0.007), XGBoost (0.039, SD = 0.006), and Random Forest (0.041, SD = 0.005), with curves closely aligned to the ideal reference line. AdaBoost, Gaussian NB, and SVC showed suboptimal calibration.

**Figure 4:**
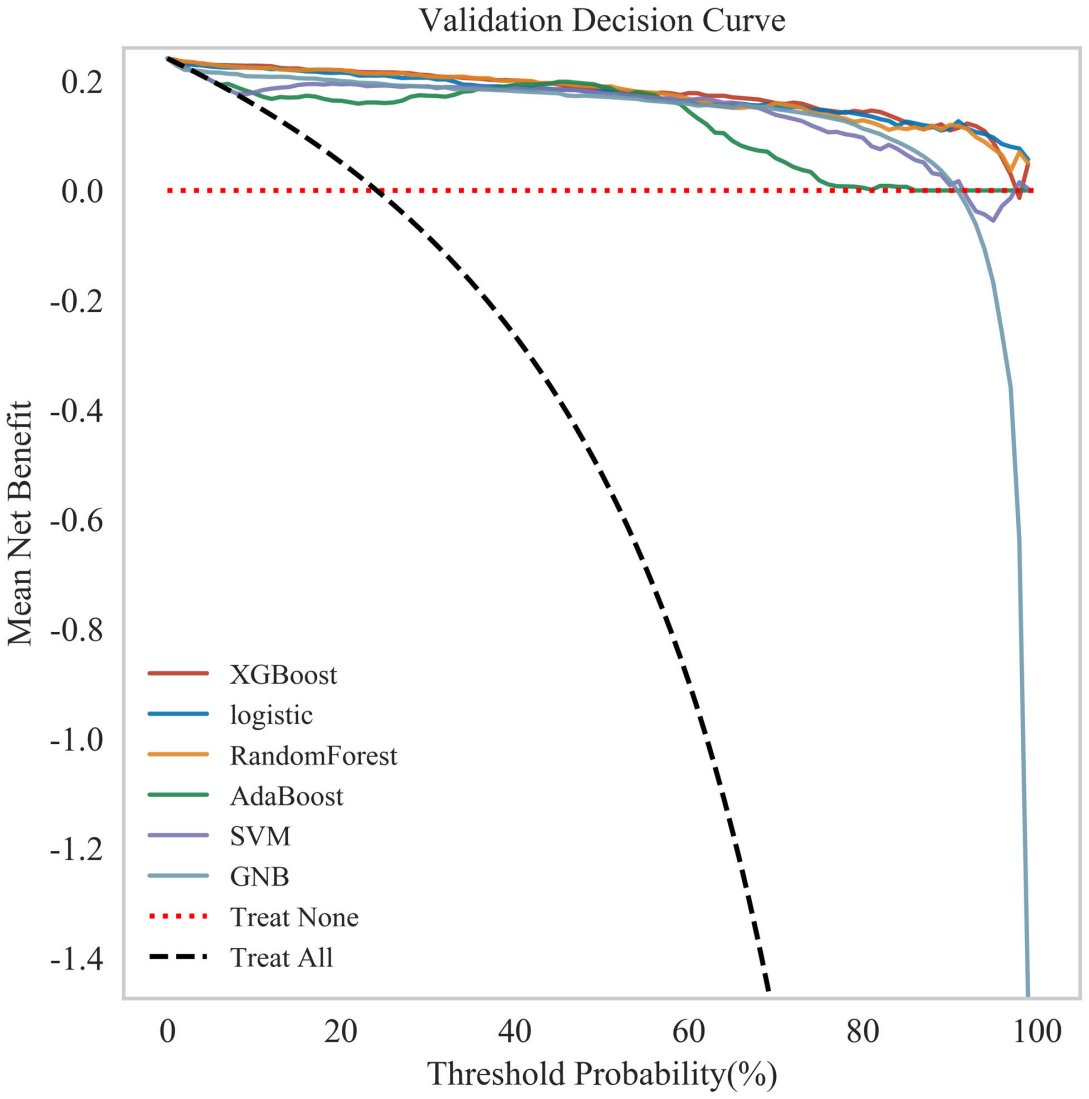
Decision curve analysis (DCA) of the six prediction models. Showed superior net benefit for Logistic regression, XGBoost, Random Forest, and SVC.

ROC curve and Calibration and decision curve analysis of six machine learning models:ROC curve analysis in (**Figure 2**). The calibration curve analysis in (**Figure 3**). Decision curve analysis in (**Figure 4**).

### 3.5 Selection of the best model algorithm.

The comprehensive predictive performance of the final Logistic regression model across the training, validation, and independent test cohorts is detailed in **Table 7**.

**Table 7:** Performance of logistic regression model for predicting DKD in the training, validation, and independent test sets.

Dataset	AUC	cutoff	Accuracy	Sensitivity	Specificity	PPV	NPV	F1-score	Kappa
Training (5-fold CV)	0.987 (0.002)	0.234 (0.101)	0.944 (0.012)	0.953 (0.026)	0.942 (0.024)	0.843 (0.052)	0.985 (0.008)	0.893 (0.018)	0.856 (0.026)
Validation (5-fold CV)	0.986 (0.004)	0.234 (0.101)	0.925 (0.024)	0.922 (0.075)	0.926 (0.044)	0.812 (0.088)	0.975 (0.022)	0.857 (0.039)	0.807 (0.055)
Test (independent)	0.979	0.22	0.917	0.897	0.924	0.788	0.966	0.839	0.783

Values in parentheses indicate standard deviation (SD). Training and validation analyses were performed via 5-fold cross-validation on 481 samples (80% of the total cohort, N = 602), with results presented as mean (SD). The independent test set included the remaining 121 samples, for which only individual performance values are reported without SD. The optimal cutoff value was 0.234 (SD = 0.101) for training and validation sets, and 0.22 for the independent test set.

AUC, area under the receiver operating characteristic curve; PPV, positive predictive value; NPV, negative predictive value.

Six machine learning models (XGBoost, Logistic regression, Random Forest, AdaBoost, SVM, and Gaussian Naive Bayes [GNB]) were evaluated on the validation set based on multiple metrics including AUC, accuracy, sensitivity, specificity, F1-score, and Kappa coefficient. Logistic regression yielded the highest AUC of 0.986 (SD = 0.004) with outstanding discriminative ability and stable performance, and it also achieved optimal sensitivity and negative predictive value for reliable identification of positive and negative samples. Considering its superior discriminative power, classification accuracy, and robustness, Logistic regression was ultimately determined as the optimal model for subsequent analyses.

**Figure 5:**
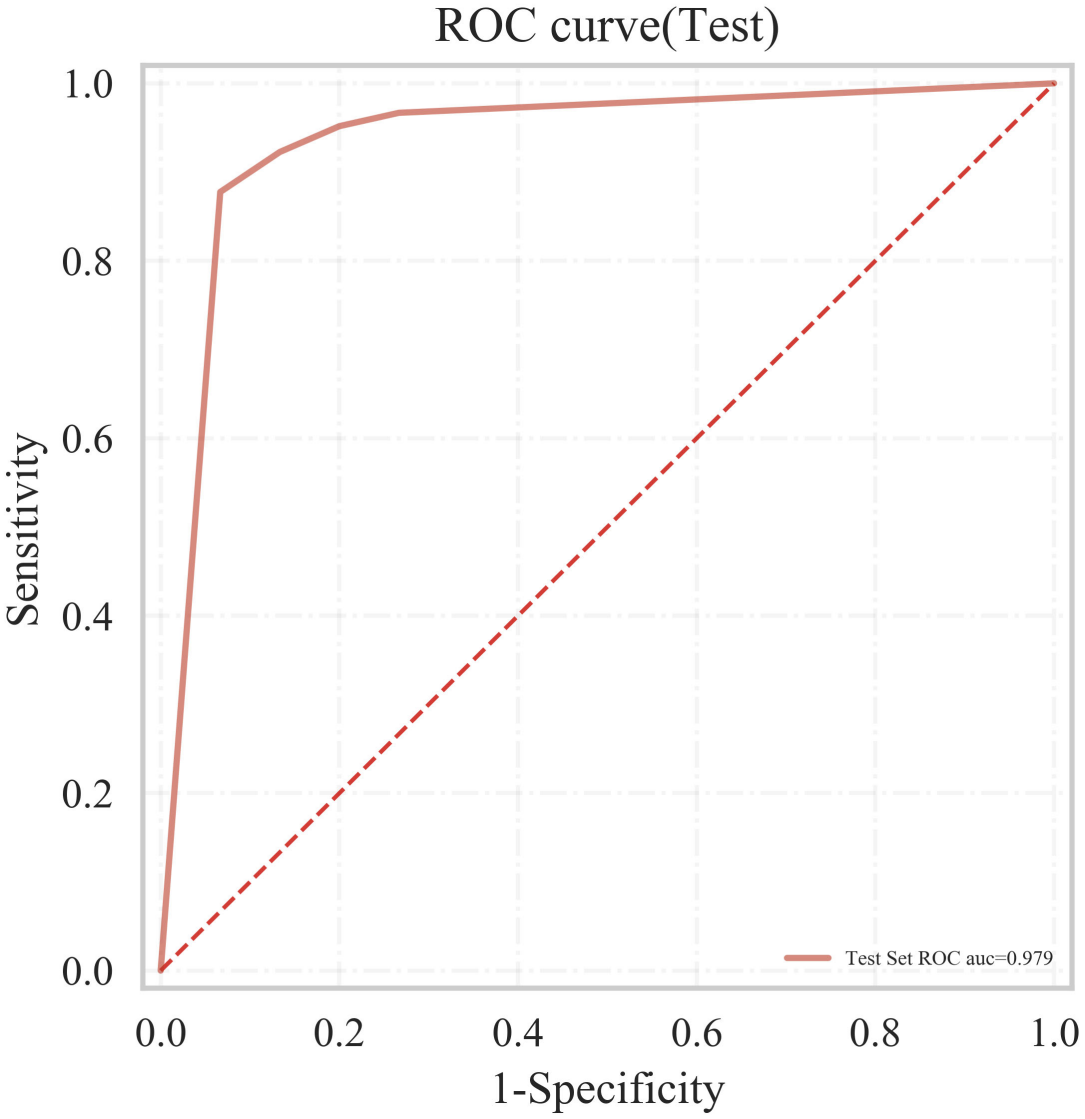
ROC curve of the logistic regression model for DKD prediction. The Logistic regression model achieved a test set AUC of 0.979, with a sensitivity of 89.7% and specificity of 92.4%, demonstrating strong predictive power for DKD.

The Logistic regression model achieved a test set AUC of 0.979, with a sensitivity of 89.7% and specificity of 92.4%, demonstrating strong predictive power for DKD (**Figure 5**).

**Figure 6:**
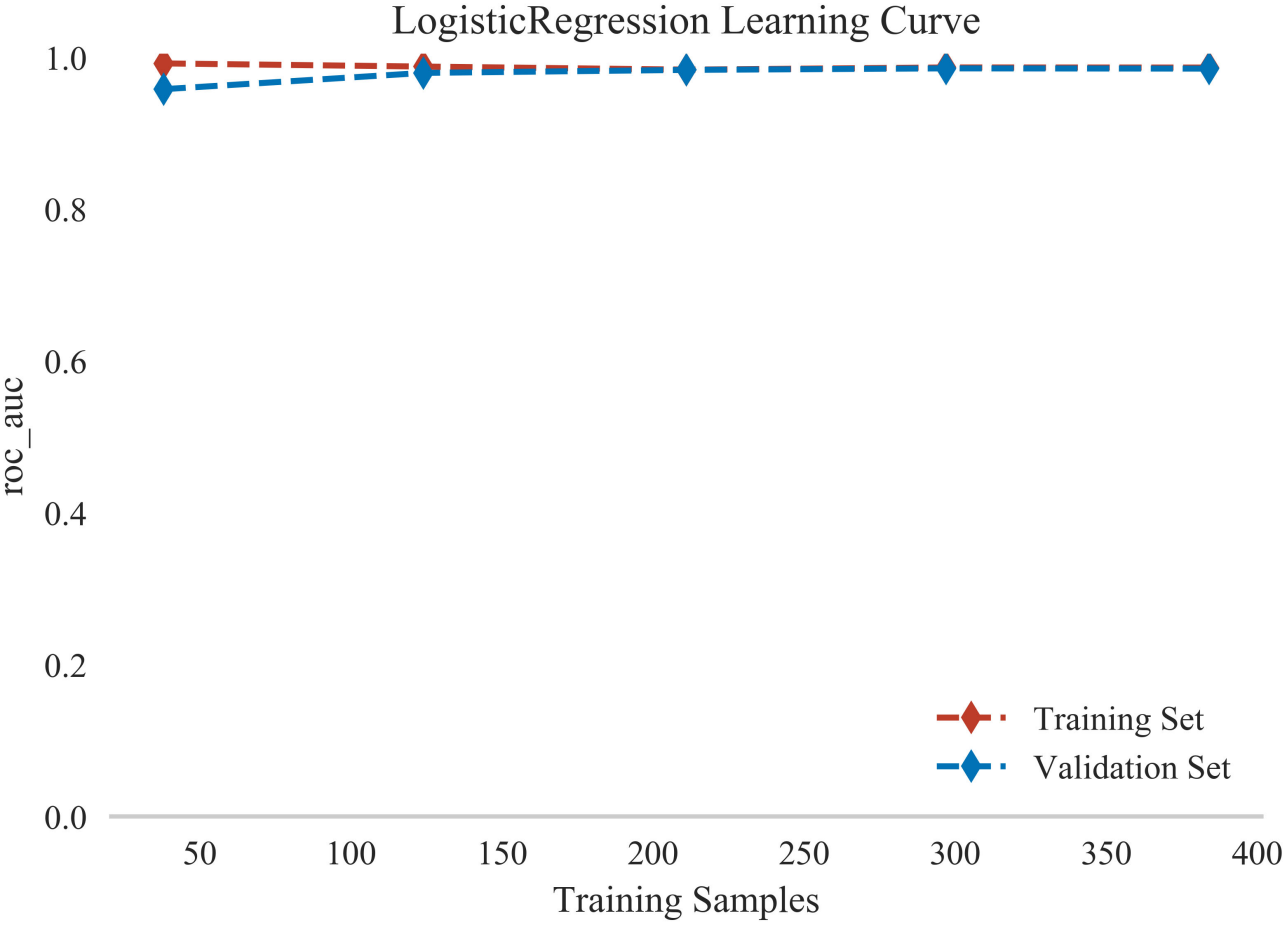
Learning curve of the logistic regression model. The learning curve of the Logistic regression model showed rapid convergence with a stable training accuracy of ~0.99 and validation accuracy of ~0.98, with a gap of only ~0.01, confirming good generalization without overfitting.

The learning curve of the Logistic regression model showed rapid convergence with a stable training accuracy of ~0.99 and validation accuracy of ~0.98, with a gap of only ~0.01, confirming good generalisation without overfitting (**Figure 6**).

**Figure 7:**
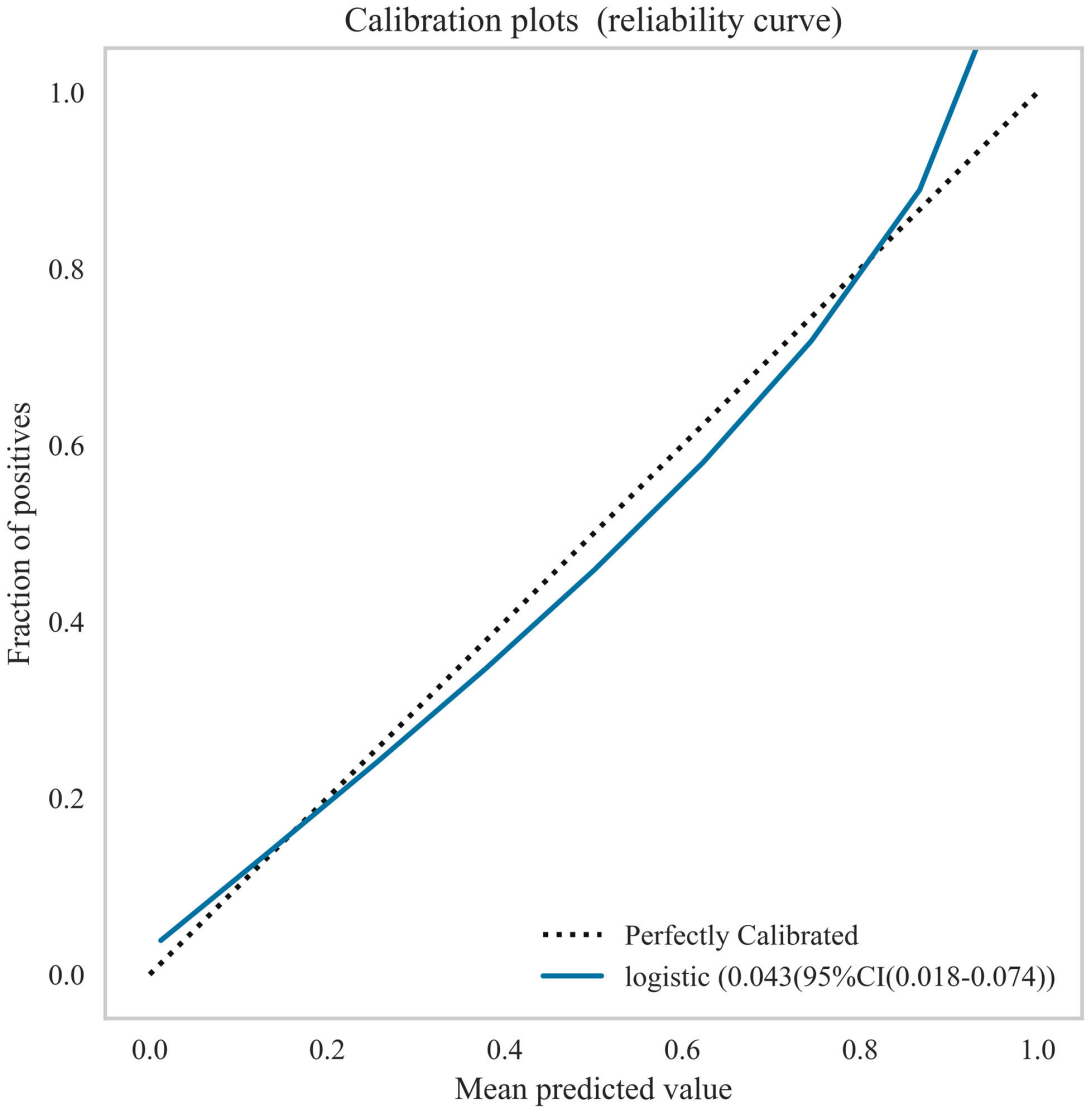
Calibration curve of the logistic regression model. The logistic regression model showed good calibration, with a Brier score of 0.043(95%CI(0.018-0.074) and a calibration curve closely following the diagonal reference line.

The Logistic regression model showed good calibration, with a Brier score of 0.043 (95% CI, 0.018-0.074) and a calibration curve closely following the diagonal reference line (**Figure 7**).

**Figure 8:**
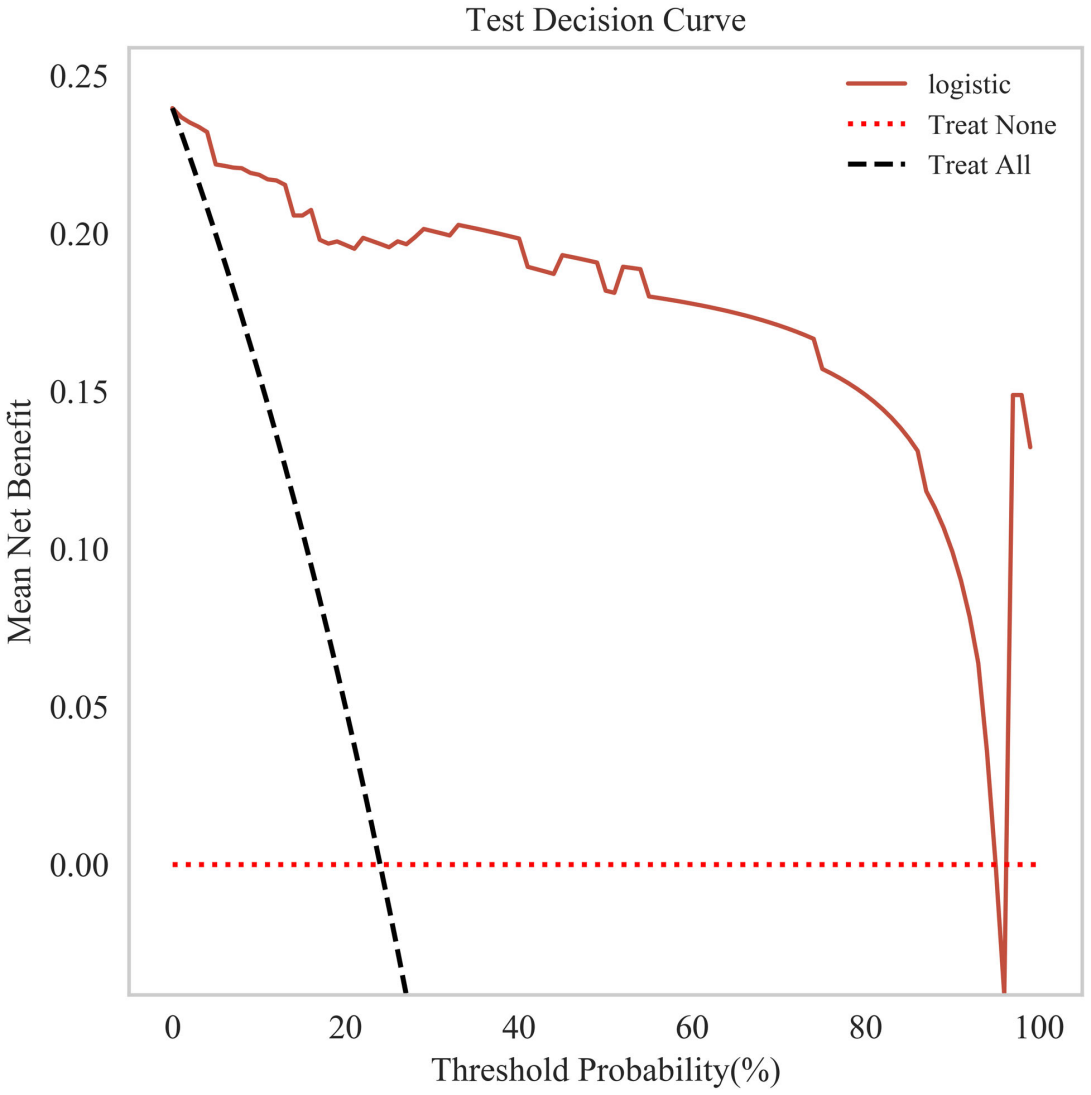
Decision curve analysis (DCA) of the logistic regression model. The logistic regression model yielded positive net clinical benefit across the threshold probability range of 0% to 95%, with its net benefit significantly superior to the two conventional clinical strategies of treating all patients and treating no patients.

The logistic regression model yielded positive net clinical benefit across the threshold probability range of 0% to 95%, with its net benefit significantly superior to the two conventional clinical strategies of treating all patients and treating no patients (**Figure 8**).

**Figure 9:**
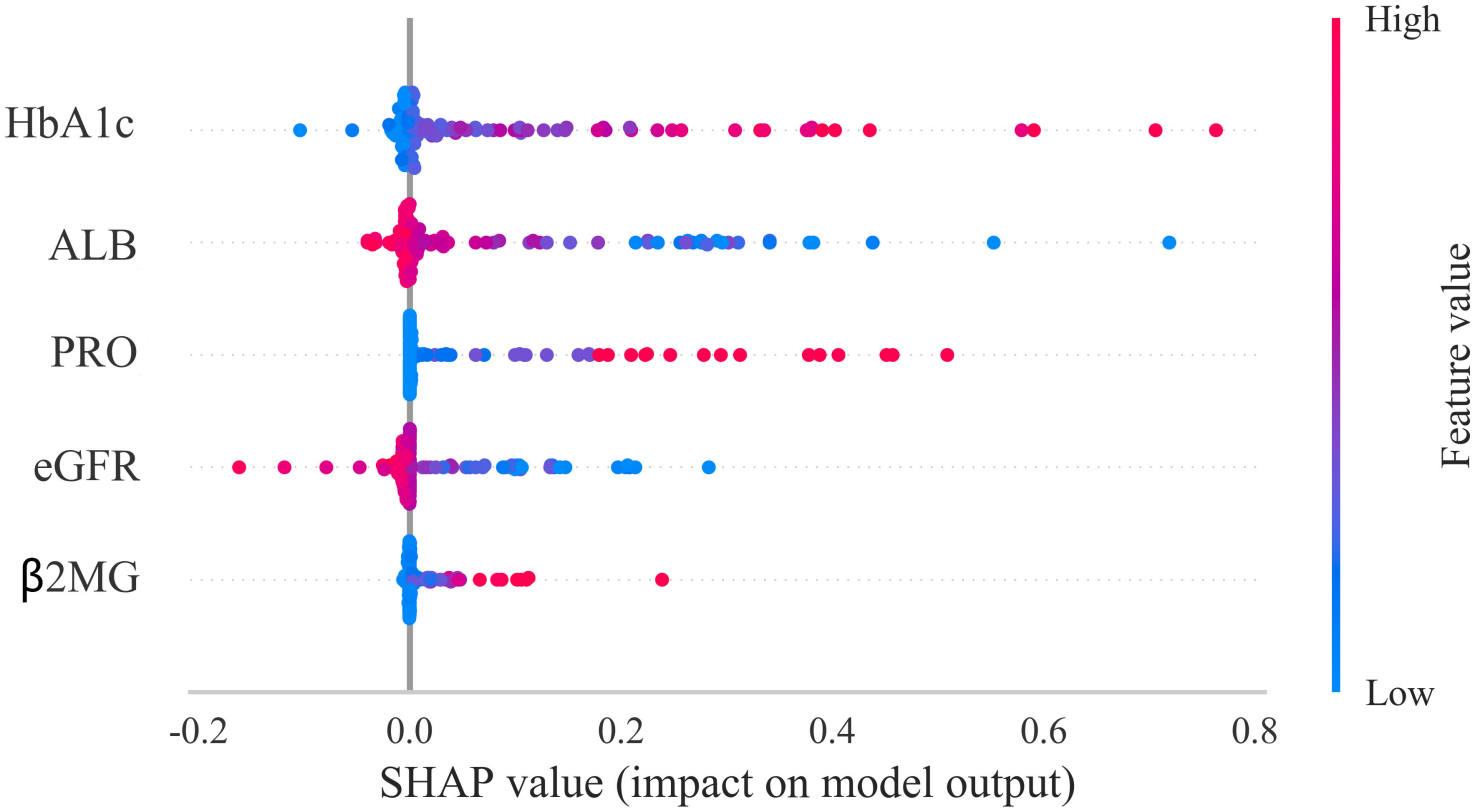
SHAP summary plot of the logistic regression model. SHAP summary bee plot for DKD prediction. Feature importance is ranked from top to bottom: HbA1c, ALB, PRO, eGFR and β_2_MG. Positive SHAP values correspond to elevated DKD risk, while negative values indicate reduced risk. The color gradient from blue to red represents increasing feature values. All feature effect directions are consistent with clinical evidence.

**Figure 10:**
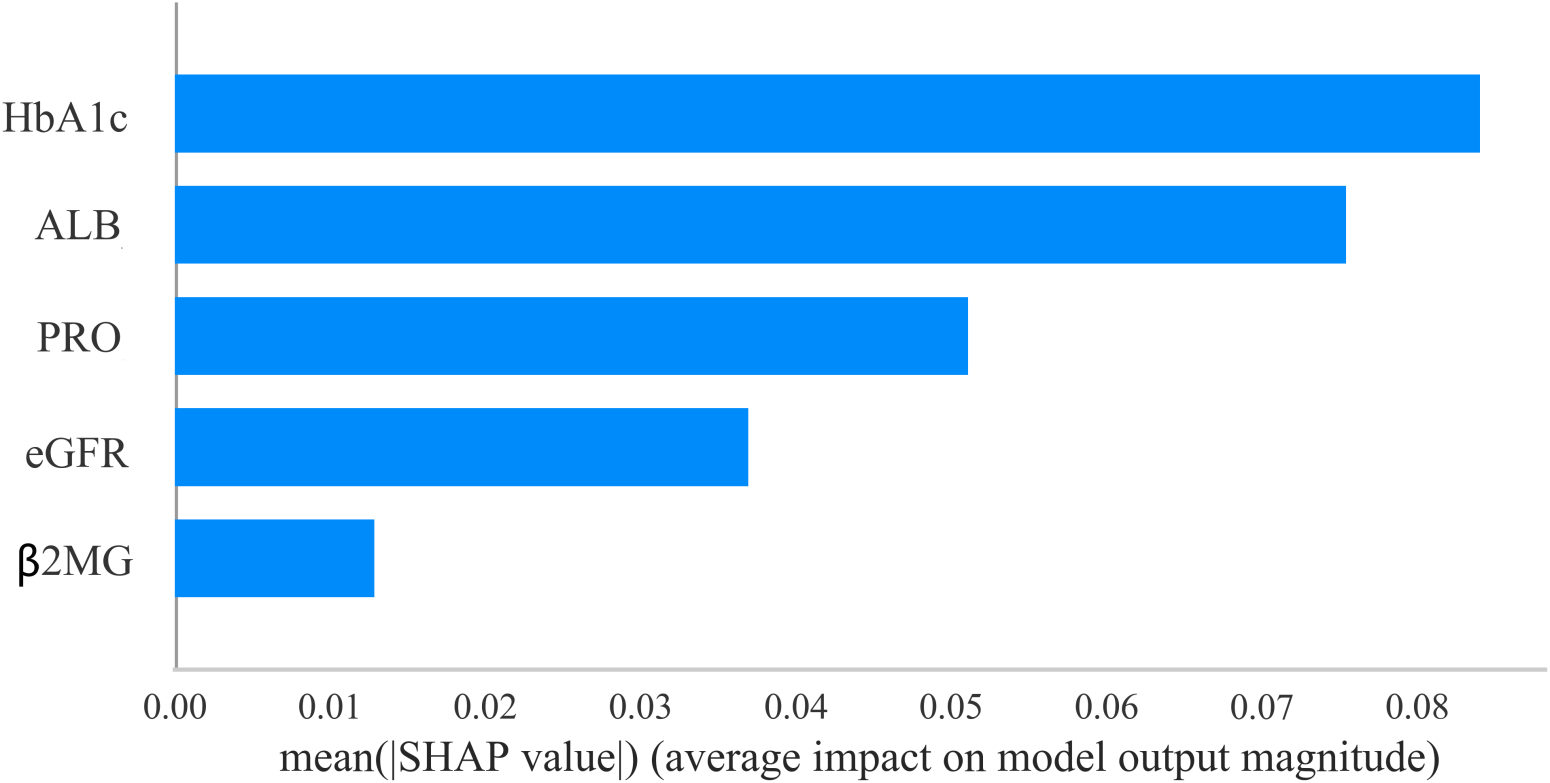
SHAP feature importance bar plot. SHAP analysis identified HbA1c, ALB, and PRO as the top three predictors for DKD according to mean absolute SHAP values, highlighting the dominant predictive roles of glycemic control and nutritional status-related indicators in DKD risk stratification.

**Figure 11:**
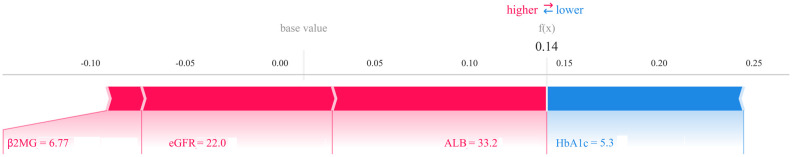
SHAP force plot for a representative patient. The SHAP force plot was used to interpret the individual predicted risk of DKD in a representative patient. Elevated HbA1c and β_2_MG acted as risk-increasing factors for DKD development, whereas higher ALB and eGFR served as protective biomarkers that reduced the risk of DKD. The cumulative predictive effect of all included clinical variables yielded a final DKD predicted probability of 0.14, demonstrating the good performance of this model in incorporating multidimensional clinical risk factors to predict DKD risk.

SHAP analysis identified HbA1c, ALB, and PRO as the top three predictors for DKD according to mean absolute SHAP values (**Figures 9**–**11**).

## 4 Discussion

In the present study, 602 patients with T2DM were included after strict screening, including 457 patients with isolated T2DM and 145 patients with DKD. Baseline comparisons showed that patients with DKD had significantly worse glucose metabolism, lipid metabolism, renal function, urinary protein, serum protein, and hematological profiles than patients without DKD. In particular, HbA1c, fasting glucose-related indicators, β_2_-microglobulin, urinary protein, and eGFR differed markedly between groups. These findings are consistent with the current understanding that DKD is driven by a combination of chronic hyperglycemia, glomerular hyperfiltration and subsequent renal function decline, tubulointerstitial injury, inflammation, oxidative stress, endothelial dysfunction, and metabolic disturbance (14, 15).

The logistic regression results further supported the biological plausibility of these associations. In univariate analysis, multiple variables were significantly associated with DKD, including SBP, FPG, 2h-PG, HbA1c, FMN, liver enzymes, lipid indicators, β_2_MG, ALB, GLB, eGFR, PRO, and HGB. After multivariable adjustment, HbA1c, creatinine-related renal injury indicators, β_2_MG, and urinary protein remained independent risk factors, whereas ALB and eGFR were protective factors. HbA1c reflects long-term glycemic exposure and has been consistently associated with the risk of microvascular complications in diabetes. Persistent hyperglycemia promotes advanced glycation end-product accumulation, oxidative stress, activation of inflammatory pathways, and glomerular basement membrane thickening, all of which contribute to DKD progression. The independent association between β_2_MG and DKD is also clinically meaningful. β_2_MG is freely filtered by the glomerulus and reabsorbed and metabolized by proximal tubular cells; increased serum β_2_MG may reflect impaired glomerular filtration and tubular injury, suggesting that tubular dysfunction may occur alongside or even before overt renal function decline in diabetic patients. Urinary protein is a classical marker of renal damage and remains central to DKD diagnosis and risk classification (10, 11). Conversely, higher eGFR and serum albumin were associated with lower DKD risk, which is consistent with preserved renal filtration capacity and better nutritional/inflammatory status (20, 21).

ROC analysis of single indicators showed that PRO and β_2_MG had the highest diagnostic performance, both with an AUC of 0.944, followed by eGFR, creatinine-related indicators, and HbA1c. These results indicate that renal injury markers and glycemic control markers are useful for identifying DKD. However, no single biomarker can fully capture the complex and heterogeneous pathophysiology of DKD (13, 14). For example, albuminuria may be absent in some patients with reduced eGFR, while some patients with albuminuria may have stable renal function for a long period (13). This heterogeneity limits the diagnostic performance of traditional single-index screening strategies and supports the development of multivariable prediction models (10, 11, 13).

Compared with single indicators and conventional regression, machine learning methods can model nonlinear relationships and complex interactions among multidimensional variables (22, 23). In this study, six machine learning models achieved excellent performance for DKD prediction. In the validation set with 5-fold cross-validation, the AUC values of XGBoost, logistic regression, random forest, AdaBoost, support vector machine, and Gaussian naive Bayes were all high, ranging approximately from 0.976 to 0.986. Among them, logistic regression showed the highest validation AUC, while XGBoost achieved balanced overall performance with high accuracy, sensitivity, specificity, positive predictive value, negative predictive value, F1 score, and Kappa value. These findings suggest that both interpretable statistical models and machine learning models can effectively integrate routine clinical variables to identify DKD risk. The strong performance of XGBoost may be related to its ability to capture nonlinear feature effects, handle feature interactions, and reduce overfitting through regularization and ensemble learning (23).

Model interpretability is a key requirement for clinical translation of machine learning algorithms (26, 27). Black-box models may achieve high accuracy but are difficult to apply in real-world decision-making if clinicians cannot understand the basis of the prediction (27). In this study, SHAP analysis was used to explain model output and quantify the contribution of individual variables (24, 25). SHAP-based interpretation can identify global feature importance and provide patient-level explanations through force plots, thereby improving model transparency. The identification of renal function indicators, urinary protein, β_2_MG, HbA1c, and serum albumin as important predictors is consistent with logistic regression and clinical knowledge, increasing confidence in the model’s reliability (10, 11, 14, 15). Such interpretable machine learning tools may help clinicians distinguish high-risk patients, prioritize further renal evaluation, and initiate individualized interventions (10–12, 26, 27).

The clinical value of this study lies in its use of non-invasive and routinely available indicators. Most predictors included in the model can be obtained from standard inpatient or outpatient laboratory tests, which reduces additional cost and improves feasibility for clinical implementation (10–12). A model based on routine clinical data may be particularly useful in primary care or endocrinology settings, where early DKD screening is essential but access to specialized nephrology evaluation may be limited (10–12). Patients identified as high risk could undergo repeated urine albumin-to-creatinine ratio testing, dynamic eGFR monitoring, individualized medication optimization with renoprotective agents including SGLT2 inhibitors and finerenone, and timely nephrology referral (10–12, 16–19). In addition, the model may support risk communication and shared decision-making by providing individualized risk estimates (26, 27).

This study also has several limitations. First, it was based on retrospective data from medical records, and selection bias and information bias cannot be fully avoided. Second, the study population was derived from a single center, which may limit generalizability to other regions, ethnic groups, community populations, or patients with different disease severity. Third, although cross-validation was used, external validation in independent multicenter cohorts is still required before clinical deployment (26, 27). Fourth, some important variables related to DKD risk, such as diabetes duration, medication history, blood pressure treatment, urine albumin-to-creatinine ratio, smoking status, inflammatory markers, and socioeconomic factors, may not have been fully incorporated (10–13). Fifth, the cross-sectional design limits causal inference; the model predicts the presence of DKD rather than the future incidence or progression of DKD. Prospective longitudinal studies are needed to determine whether the model can predict new-onset DKD, rapid eGFR decline, albuminuria progression, or renal endpoints (26, 27).

## 5 Conclusion

This study developed and evaluated non-invasive prediction models for DKD in patients with T2DM using routine clinical and laboratory indicators. HbA1c, β_2_MG, urinary protein, eGFR, serum albumin, and renal function-related parameters were closely associated with DKD. Machine learning models, particularly XGBoost and other ensemble algorithms, showed excellent predictive performance and good clinical interpretability when combined with SHAP analysis (23–25). These findings suggest that interpretable machine learning based on routine clinical data may serve as a practical tool for early DKD risk screening and individualized management (10–12, 26, 27). Future studies should validate the model in larger prospective multicenter cohorts and evaluate its impact on clinical decision-making and renal outcomes (26, 27).

## Data Availability

The datasets presented in this study can be found in online repositories. The names of the repository/repositories and accession number(s) can be found in the article/supplementary material.
